# Temporal trends in severe malaria in Chittagong, Bangladesh

**DOI:** 10.1186/1475-2875-11-323

**Published:** 2012-09-12

**Authors:** Richard James Maude, Mahtab Uddin Hasan, Md Amir Hossain, Abdullah Abu Sayeed, Sanjib Kanti Paul, Waliur Rahman, Rapeephan Rattanawongnara Maude, Nidhi Vaid, Aniruddha Ghose, Robed Amin, Rasheda Samad, Emran Bin Yunus, M Ridwanur Rahman, Abdul M Bangali, M Gofranul Hoque, Nicholas PJ Day, Nicholas J White, Lisa J White, Arjen M Dondorp, M Abul Faiz

**Affiliations:** 1 Mahidol-Oxford Tropical Medicine Research Unit, Faculty of Tropical Medicine, Mahidol University, Bangkok, Thailand; 2 Centre for Tropical Medicine, Nuffield Department of Clinical Medicine, Churchill Hospital, Oxford, UK; 3 Department of Infection and Tropical Medicine, Heartlands Hospital, Birmingham, UK; 4 Chittagong Medical College Hospital, Chittagong, Bangladesh; 5 The Royal London Hospital, Whitechapel, London, UK; 6 Dhaka Medical College, Dhaka, Bangladesh; 7 Centre for Specialized Care and Research, Chittagong, Bangladesh; 8 Dev Care Foundation, Dhaka, Bangladesh; 9 Shaheed Shwarwardhy Medical College, Dhaka, Bangladesh; 10 World Health Organization, Country Office, Dhaka, Bangladesh

**Keywords:** Malaria, Bangladesh, Epidemiology, Incidence, Severe, Falciparum, Vivax

## Abstract

**Background:**

Epidemiological data on malaria in Bangladesh are sparse, particularly on severe and fatal malaria. This hampers the allocation of healthcare provision in this resource-poor setting. Over 85% of the estimated 150,000-250,000 annual malaria cases in Bangladesh occur in Chittagong Division with 80% in the Chittagong Hill Tracts (CHT). Chittagong Medical College Hospital (CMCH) is the major tertiary referral hospital for severe malaria in Chittagong Division.

**Methods:**

Malaria screening data from 22,785 inpatients in CMCH from 1999–2011 were analysed to investigate the patterns of referral, temporal trends and geographical distribution of severe malaria in Chittagong Division, Bangladesh.

**Results:**

From 1999 till 2011, 2,394 malaria cases were admitted, of which 96% harboured *Plasmodium falciparum* and 4% *Plasmodium vivax*. Infection was commonest in males (67%) between 15 and 34 years of age. Seasonality of malaria incidence was marked with a single peak in *P. falciparum* transmission from June to August coinciding with peak rainfall, whereas *P. vivax* showed an additional peak in February-March possibly representing relapse infections. Since 2007 there has been a substantial decrease in the absolute number of admitted malaria cases. Case fatality in severe malaria was 18% from 2008–2011, remaining steady during this period.

A travel history obtained in 226 malaria patients revealed only 33% had been to the CHT in the preceding three weeks. Of all admitted malaria patients, only 9% lived in the CHT, and none in the more remote malaria endemic regions near the Indian border.

**Conclusions:**

The overall decline in admitted malaria cases to CMCH suggests recent control measures are successful. However, there are no reliable data on the incidence of severe malaria in the CHT, the most endemic area of Bangladesh, and most of these patients do not reach tertiary health facilities. Improvement of early treatment and simple supportive care for severe malaria in remote areas and implementation of a referral system for cases requiring additional supportive care could be important contributors to further reducing malaria-attributable disease and death in Bangladesh.

## Background

Over a million suspected cases of malaria are reported annually to the World Health Organization (WHO) in Bangladesh, although the number of confirmed cases is only 60,000, and estimates of the true incidence vary widely [1-3]. Cases from most medical college hospitals, specialized hospitals, NGO hospitals and private clinics and hospitals are not included and the true number is estimated to be three times higher [1,4]. Most malaria cases in Bangladesh did not have a confirmatory blood test until recently and clinical diagnosis of malaria is known to be unreliable [5,6]. Since 2000, no clear decrease in the overall annual number of cases has been documented, but this could be related to counting methods [2,3,5,7]. From 2006–2008, the number of people tested for malaria more than doubled [5], but despite this, in 2008 only a third of 1.3 million reported suspected cases had a blood test for malaria (microscopy or rapid diagnostic test), one fifth of which (85,000) were positive [3]. The true incidence of malaria is thus probably in the range of 150,000-250,000 cases per year [1,3,4].

It is likely that an increase in the efficiency of diagnosis and reporting has masked a decline in incidence. Access to artemisinin combination therapy (ACT) has doubled since 2005 [3] and annual reported numbers of confirmed deaths from malaria has decreased 10-fold since 2005 to 47 in 2009 [5]. It should be noted that these figures do not include deaths in many hospitals and private facilities or those that do not reach health care and the true figure is likely to be much larger [4].

**Figure 1:**
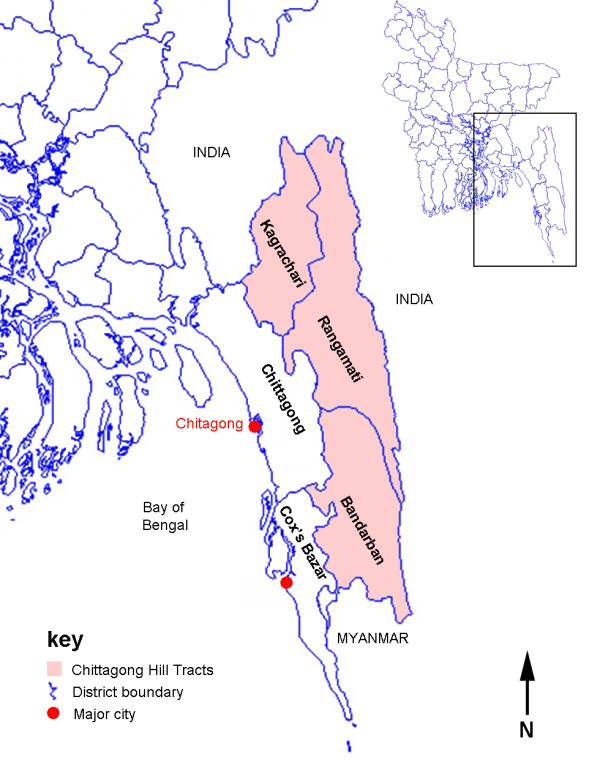
Map of Chittagong division.

Published data on geographical distribution of malaria cases in Bangladesh are patchy and incomplete. Under-reporting is thought to be a particular problem in remote areas [8]. The first national malaria prevalence survey in Bangladesh was undertaken in 2007. Over 85% of all cases and 95% of severe cases were reported to occur in the south of Chittagong Division, in 5 Districts: Bandarban, Chittagong, Cox’s Bazar, Khagrachari and Rangamati (Figure 1) [8,9]. Malaria transmission in this area is highly seasonal with an estimated annual rate of infection of around 8 per 1000 people at risk [1,10]. The highest rates of malaria transmission are thought to be in the Chittagong Hill Tracts (CHT) i.e. Bandarban, Khagrachari and Rangamati Districts which together account for 80% of cases [7-9]. These have very low population density and are inland, mostly forested hilly areas.

Accurate figures for numbers of cases of severe malaria in Bangladesh are not readily available. An exception is a WHO report from 2006 reporting 513 case fatalities out of a total of 51,705 confirmed falciparum malaria cases, of which 3,539 were severe [8]. It was estimated that there were 2,200-12,000 actual fatal cases in that year and 1,108,000-6,677,000 total cases of all species malaria in Bangladesh in 2006 [2]. A later WHO report stated there were 1,320,581 reported cases in 2006 [3]. With a case fatality rate in severe malaria of 15-20% [11], the number of fatal cases suggests a total of 11,000-80,000 severe malaria cases in that year. More recently, a total number of 3591 cases of severe malaria cases were counted between July 2008 and May 2009 [7]. There are no published data on long-term trends and very little on the geographical distribution of severe malaria in Bangladesh [7].

Mortality from severe malaria can be greatly reduced by intravenous anti-malarials (particularly artesunate) and high quality supportive care [11]. In Bangladesh, intravenous anti-malarials are widely available in local hospitals but access to advanced supportive care (e.g. blood transfusion, mechanical ventilation and renal dialysis) is much more limited. Recommended practice in Bangladesh is for all severe cases of malaria to be treated in hospital [12]. Numbers of malaria admissions may thus be a useful surrogate in the absence of reliable data on severe disease. Numbers of patients with malaria admitted to hospital in Bangladesh have been published since 2007. There were 5678 such admissions reported in 2007, 3042 in 2008 and 3287 in 2009 [5]. This is around half of the lowest estimate for the annual number of severe cases (above) and very few of these would have had access to tertiary level care. There is thus likely to be a large burden of patients who do not reach hospital, representing potentially preventable mortality. In order to improve care for people with severe malaria in Bangladesh it is important to describe this population in more detail.

A study was undertaken using routinely collected malaria screening data from the past 13 years from Chittagong Medical College Hospital (CMCH), Bangladesh to investigate the patterns of referral of patients, temporal trends and geographical distribution of severe malaria in Chittagong Division.

## Methods

### Place and period of study

The study was conducted at CMCH, Chittagong, Bangladesh from January 1999 to December 2011. CMCH is a government-run 1000-bed teaching hospital and the main tertiary referral hospital for severe malaria in Chittagong Division. CMCH receives referrals from throughout southeast Bangladesh, particularly those severe cases who require more intensive management as it is the only government hospital in the south of Chittagong Division with facilities for intensive care and haemodialysis. Its patients are mostly in the lower income range. There is a high quality malaria diagnostic service on-site with a well-developed recording system for malaria screening results. As the malaria laboratory is readily accessible, being located next to the medical wards, and the test is free to the patients, most if not all patients with suspected malaria undergo testing through this facility. The vast majority of malaria patients admitted to CMCH have severe disease. The results thus provide a representative picture of severe malaria cases admitted to CMCH over time. Assuming referral patterns from outlying clinics and hospitals to CMCH have not changed significantly in this period, they may also give an indication of long-term trends of severe malaria across Chittagong Division.

Results of all screening of inpatients for malaria by the CMCH malaria diagnostic laboratory during this period were collated and analysed. Malaria diagnosis was by microscopy of thick and thin blood films. Date of testing, age, gender and smear results were collected for all malaria positive patients throughout, area of residence from 2002 (District 2002–2007, District and Thana (i.e. subdistrict) 2008–2011), and all of these from 2008 onwards for smear negative patients. In 2006–2011, additional data were collected from patients with malaria and their relatives to determine whether patients had travelled to another Thana in the 3 weeks before presentation. Admission GCS and outcome were also recorded during this period. Many of the malaria slide positive patients were enrolled in a series of clinical studies of severe malaria and a detailed clinical description will be presented elsewhere. Data on population density were taken from the 2001 national census [13] and data on age distribution from the 2004 Sample Vital Registration Survey [14]. Rainfall data for Chittagong were provided by the Bangladesh Meteorological Department, Government of Bangladesh.

## Results

In total, there were 22,785 inpatients screened for malaria at CMCH between January 1999 and December 2011. Of these, 2,394 (11%) were positive, 2295/2,394 (96%) with *P. falciparum* (mean of 177 cases per year), and 93/2,394 (4%) *P. vivax*. One patient (1/2,384 (0.04%)) had *Plasmodium malariae*. The median (IQR) age of those who were parasite negative was 25 (15–45) years and those with *P. falciparum* 26 (18–40) years, p = 0.51. Of those with *P. falciparum*, 67% were male whereas 59% of parasite negative patients were male (p = 0.002). For 251 unselected patients with *P. falciparum* between 2008 and 2011, admission Glasgow Coma Scale and outcome were recorded. GCS was <11 in 103/251 (41%) and 44/251 (18%) died.

**Figure 2:**
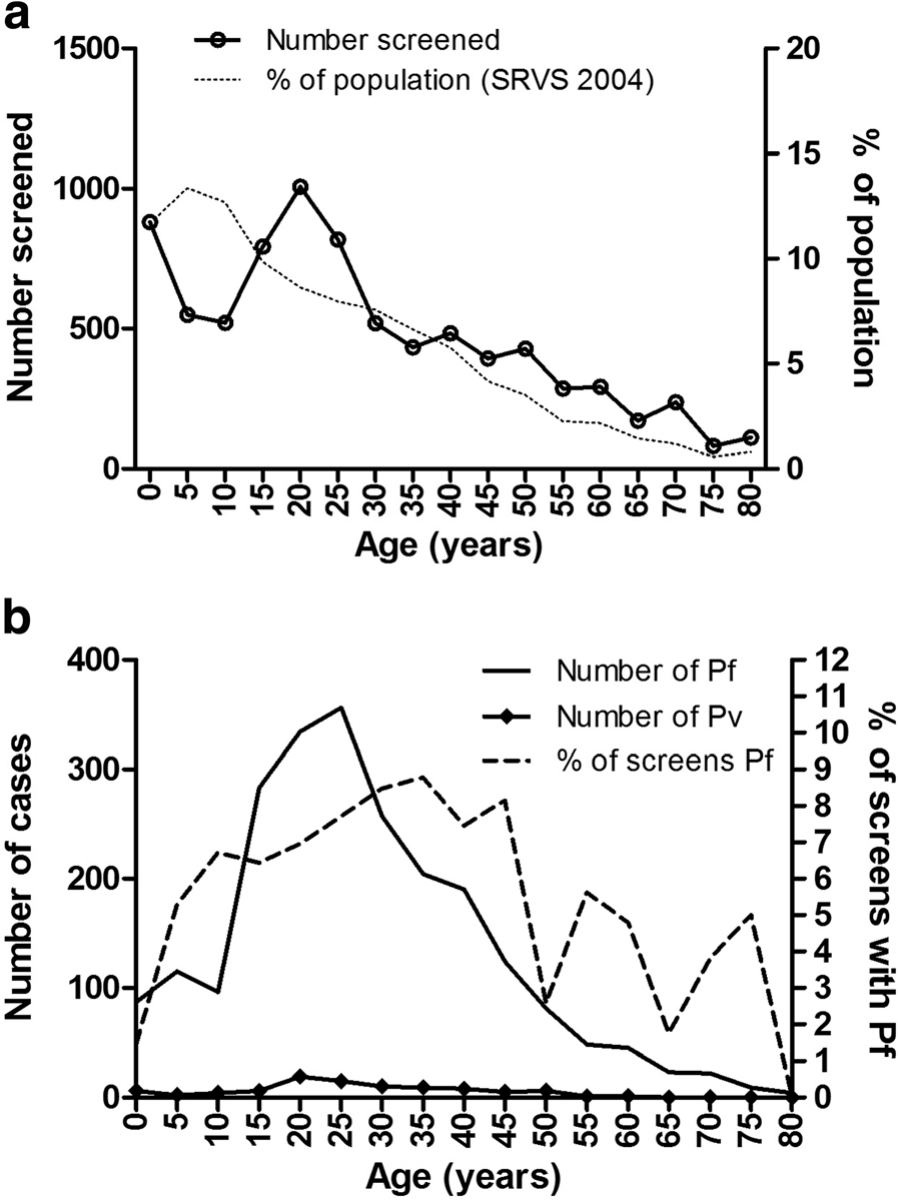
**Age profiles in 5 year age groups.** (**a**) screened patients, (**b**) *P. falciparum* (Pf) and *P. vivax* (Pv) positive patients and C percent of total deaths and percent of total survivors. The solid lines are numbers of individuals and the broken lines are percentages. % of population is the percentage of the population in that age group in the 2004 Sample Vital Registration Survey (SRVS) [14].

The proportions of patients screened for malaria broken down by age group roughly mirrored the structure of the general population (Figure 2A). There were two exceptions to this. Those age 5–19 years were under-represented and those age 20–29 years were over-represented. *Plasmodium falciparum* was commonest in those aged 15–34 years and the highest proportion positive was in those age 35‐39 years (Figure 2B). Of those with *P. falciparum*, 24.0% were age 18 years or less. Few *P. falciparum* positive patients were ≤10 years (11.6% of positives versus 19.5% of those screened)) or over 50 years old (10.1% of positives versus 16.3% of those screened). The age profile of those who died was no different to that of those who survived (Figure 2C).

### Temporal trends

**Figure 3:**
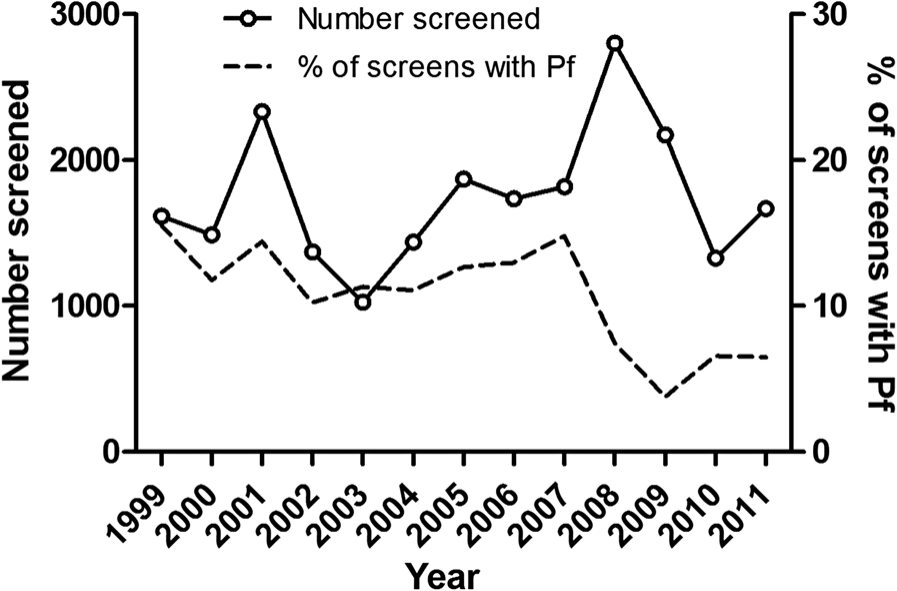
**Long term trends.** A annual number of individuals screened for malaria and % positive for *P. falciparum* from 1999–2011. B Annual numbers of individuals positive for *P. falciparum* (Pf) and *P. vivax* (Pv). C Median (IQR) age for those with *P. falciparum* from 1999–2011. D Annual number and % of individuals with *P. falciparum* from CHT 2002–2011 (data on area of residence were not collected before 2002).

The annual number of patients screened for malaria at CMCH was highest in 2008–2009 (Figure 3A). Both the number and proportion that were positive for *P. falciparum* decreased dramatically from 2007 onwards (Figures 3A and B). The number with *P. vivax* decreased from 1999 (Figure 3B). The median age of people with *P. falciparum* infection did not change from 1999–2011 (Figure 3C). The proportion of patients with *P. falciparum* resident in the CHT increased from 2008, although the absolute number did not change (Figure 3D).

### Seasonality

**Figure 4:**
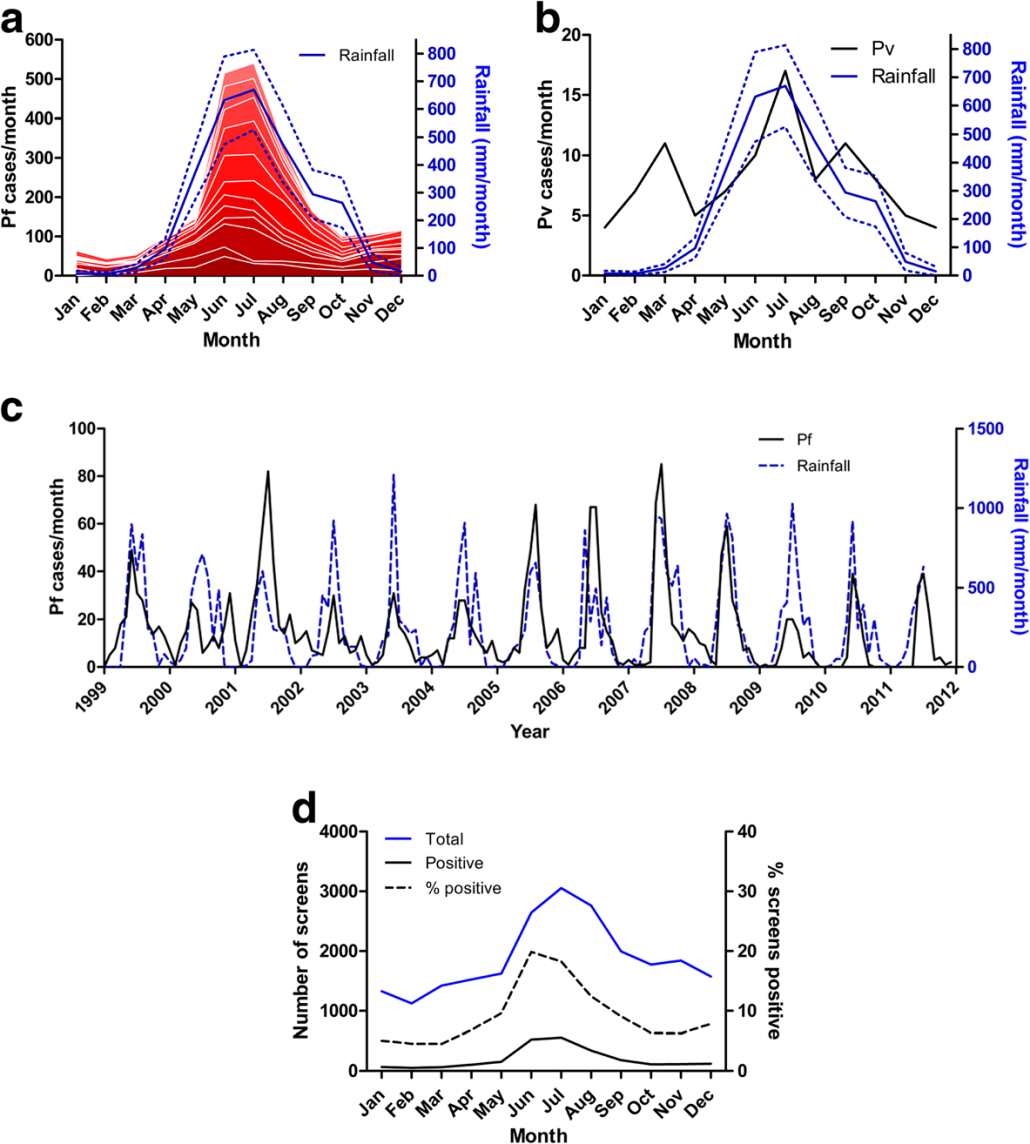
**Seasonality.** Cumulative numbers of monthly cases of *P. falciparum* (Pf) (**a**) and *P. vivax* (Pv) (**b**) at CMCH and rainfall from 1999 to 2011 (shown as average with 95% confidence interval). In **a**, the bottom band is 1999 and the top band 2011. **c** Cases of *P. falciparum* each month with monthly rainfall. **d** Monthly cumulative numbers screened for malaria with proportion and number positive for *P. falciparum.*

The number of *P. falciparum* positive cases was highest from June to August and this was highly consistent from 1999 to 2011 (Figure 4A). There was a clear association with the timing of peak rainfall (Figure 4A). The amount of rainfall per month correlated with the number of malaria cases in the same month (p < 0.0001, R^2^ = 0.40), consistent with a peak of malaria cases in the wet season (Figure 4C). However, the annual amount of rainfall and the total in the wettest three months (June-August) were unrelated to the number of cases of malaria in the same or the following month. There was a different seasonal pattern in *P. vivax* caseload, with a broader peak and an additional peak in February to March 1999 (Figure 4B). During the peak transmission season, both the total number of patients screened and the proportion of those that were positive for *P. falciparum* increased (Figure 4D). The amplitude of seasonal variation of *P. falciparum* was around 80-90%.

### Geographical distribution

**Figure 5:**
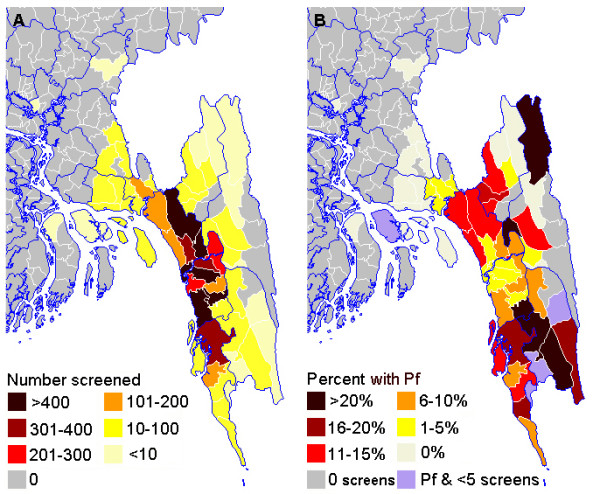
**Geographical distribution of malaria.A** patients screened for malaria at CMCH 2008–2011 shown as number per Thana. **B** Percent of those screened in each Thana who were positive for *P. falciparum*. Where a Thana had case(s) of *P. falciparum* but less than 5 individuals were screened, percentages are not shown. Thana boundaries are white and District boundaries blue.

**Figure 6:**
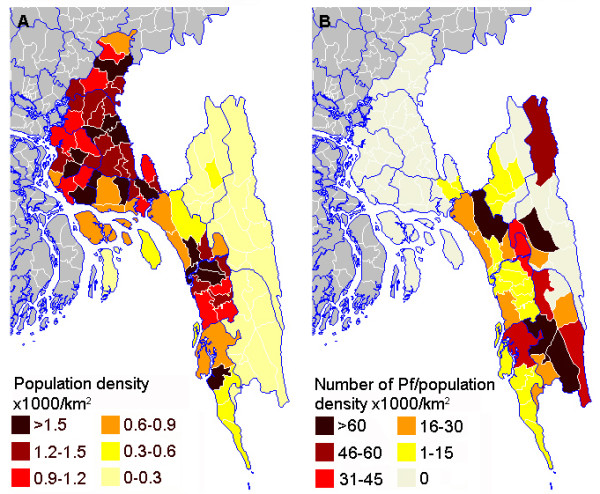
**Population density and malaria.** (**A**) Population density in Chittagong Division from the 2001 census [13]. The CHT are the three Districts in pale yellow on the right. (**B**) Number of *P. falciparum* malaria cases seen at CMCH 2008–2011 per 1000 population km^-2^. Thana boundaries are white and District boundaries blue.

An animated map of the annual numbers of *P. falciparum* malaria cases by area of residence from 2002 to 2011 is shown in Additional file 1. The numbers of malaria cases from each District are summarized in Table 1. The geographical distributions of patients screened and positive for malaria are shown in Figures 5 and 6. In Figure 6A the three Districts in pale yellow on the right are the CHT. Overall, 400/7950 (5%) screened patients and 57/484 (12%) malaria positive patients (96% *P. falciparum*) were resident in the CHT in 2008–2011. 86/1147 (7.5%) malaria positive patients from 2002–2007 were resident in the CHT. The annual number of malaria cases who lived in the CHT varied from 2002–2011 although numbers were small and there was no clear overall trend (Figure 3C).

**Table 1:** **Number screened, *P. falciparum *and *P. vivax *positive cases by District of residence from 2008-2011**

**District**	**Screened (%)**		**Pf (%)**		**Pv (%)**		**% of screens positive**
Chittagong	6576	(82.7%)	310	(65.0%)	4	(50.0%)	5%
Cox's Bazar	696	(8.8%)	91	(19.1%)	1	(12.5%)	13%
Bandarban	153	(2.0%)	28	(5.9%)	1	(12.5%)	19%
Khagrachari	100	(1.3%)	8	(1.7%)	1	(12.5%)	9%
Rangamati	147	(1.8%)	19	(4.0%)	0	(0.0%)	13%
Feni	156	(1.9%)	13	(2.7%)	1	(12.5%)	9%
Other (Chittagong Division)	61	(0.8%)	0	(0.0%)	0	(0.0%)	0%
Other (other Divisions)	11	(0.1%)	3	(0.6%)	0	(0.0%)	27%
Unknown	50	(0.6%)	5	(1.0%)	0	(0.0%)	10%
**Total**	**7950**		**477**		**8**		**6%**

### Travel

In 2006 to 2011 travel in the 3 weeks before admission was recorded for 266 unselected patients with severe malaria (Table 2). All patients surveyed were resident in Chittagong Division, 41/266 (15%) of whom lived in the CHT. A total of 74/266 (28%) had visited another Thana in the preceding 3 weeks of whom 47/74 (64%) had visited the CHT but did not live there. Thus, in total, 88/266 (33%) had been in the CHT in the preceding 3 weeks. All patients who travelled to Thana in the CHT were resident outside of the CHT. 10/266 (3.8%) of the patients lived in Chittagong City and had not travelled, thus suggesting there is malaria transmission in the city itself.

**Table 2:** Travel to another Thana within the 3 weeks preceding admission

**Place**	**Travelled**				**Did not travel**	
	**Destination**		**Residence**		**Residence**	
**Chittagong District**	**20**	**(27.0%)**	**45**	**(60.8%)**	**104**	**(54.2%)**
Chittagong city	5	(6.8%)	36	(48.6%)	10	(5.2%)
Other	15	(20.3%)	9	(12.2%)	94	(49.0%)
**Cox's Bazar District**	**2**	**(2.7%)**	**13**	**(17.6%)**	**36**	**(18.8%)**
**Chittagong Hill Tracts**	**47**	**(63.5%)**	**0**	**(0.0%)**	**41**	**(21.4%)**
Bandarban District	19	(25.7%)	0	(0.0%)	19	(9.9%)
Khagrachari District	8	(10.8%)	0	(0.0%)	8	(4.2%)
Rangamati District	10	(13.5%)	0	(0.0%)	14	(7.3%)
Thana not specified	10	(13.5%)	0	(0.0%)	0	(0.0%)
**Unknown**	**5**	**(6.8%)**	**16**	**(21.6%)**	11	(5.7%)
**Total**	**74**		**74**		**192**	

Responses to the question “did you travel anywhere within the previous 3 weeks?” for 266 patients with *P. falciparum* malaria from 2006–2011 and Thana of residence for those who did and did not travel.

## Discussion

The present analysis strongly suggests that only a small proportion of severe cases reach tertiary care (0.2-1.6%), since a mean of 177 malaria patients per year were admitted to CMCH, the only tertiary care facility in the area, whereas the estimated total numbers of severe cases nationally is around 11,000-80,000 per year of which 80% reside in the CHT. One of the explanations could be the financial cost of hospitalization, which is high in proportion to the average income in Bangladesh and can thus be a strong disincentive against admission of less severe patients. Many of the less severe cases we probably treated in peripheral Thana or District hospitals and many cases of severe malaria probably remained untreated in the community. Of the patients admitted to CMCH, 96% had *P. falciparum* and 4% of patients had *P. vivax*, whereas mortality was only associated with *P. falciparum*. Previous studies in uncomplicated malaria in this region show that 70% of malaria is caused by *P. falciparum* and 30% by *P. vivax*[1,5,8], which emphasizes the benign nature of *P. vivax* infections in this region. Malaria infection was commonest in young adult males in agreement with previous data from the south of Chittagong Division [1]. This may be related to greater occupational exposure of this group to forest malaria [15]. As the breadwinners, it could be postulated that they are also the family member most likely to be supported to attend a distant hospital for potentially expensive treatment [16], hence their over-representation in those screened for malaria.

The number of hospitalized *P. falciparum* cases at CMCH decreased since 2007, despite an increase in the number of patients screened. This decrease was particularly dramatic from 2008–2010. During this period, there have been a number of changes in malaria programmes in the community and a greater than five-fold increase in funding for malaria control [3,10]. A large increase in the numbers of patients who receive early antimalarial treatment has occurred in the feeder hospitals and clinics referring to CMCH. In particular there has been a doubling of ACT usage from 2007 to 2008 [3,10] and an increase in the availability of parenteral antimalarials. In addition, a new large-scale programme of free distribution of insecticide-treated bednets [3,10] and introduction of rapid diagnostic tests in the community began in 2008 [3,10]. The present study adds to the evidence that these strategies are having a significant impact. However, potential pockets of high transmission, which are mostly in remote areas [15] may not have been sufficiently covered by this study. Over the past two years there have been initiatives aiming at increased availability of early treatment in more remote areas, including early intravenous therapy in the District hospitals, and as a result it is possible a smaller proportion of cases are being referred to tertiary hospitals.

There was a strong and consistent seasonal pattern of *P. falciparum* incidence, with a large peak between May and September each year largely coinciding with the maximum rainfall during the monsoon season (June-August). This finding is in contrast with the pattern reported in two earlier reports [3,6] describing one transmission peak in March-May and one in September-November, with June-August being described as ‘off-peak months’ [3]. However, in both these publications no monthly incidence data were presented. For the incidence of *P. vivax* an additional peak was observed in the months from February to March, before arrival of the rains and transmission of *P. falciparum*, which uses the same vector system. The same seasonality has been described in vivax malaria in Hooghly District in West Bengal in India, and suggests that these are long latency relapse cases of vivax malaria [17].

By far the majority of individuals screened for malaria were from the south of Chittagong Division with relatively few from the CHT. All the malaria positive cases were from the south of Chittagong Division in the five endemic Districts: Chittagong, Cox’s Bazar, Kagrachari, Rangamati and Bandarban. Over 80% of cases in Bangladesh are thought to be resident in the CHT [7-9], although in this study only 12% of those referred to CMCH lived there. This is despite it being the main referral hospital for those needing more advanced care and local policy being to refer the sickest cases from all other government hospitals in the area to CMCH.

As expected from existing epidemiological data [9], the probability of having malaria was highest in screened patients coming from the CHT, particularly the southern part: Lama and Alikadam Thana in Bandarban District but also adjacent Lohagara in Chittagong District. The high rate in Lohagara was mainly caused by an apparent focal epidemic of *P. falciparum* in 2010.

Population density in the CHT is much lower than elsewhere in Chittagong Division. The most densely inhabited areas are Chittagong City and Cox’s Bazar and their surroundings and both these areas had very few malaria cases. The highest malaria positivity rates per population density in this study were mostly in those from areas of low population density in a band from north to south through the centre of Chittagong Division. These were Fatikchari and Rangonia in Chittagong District, Kawkhali and Rangamati Sadar in Rangamati District and Bandardan Sadar, Lama, Alikadam, and Thanchi Thana in Bandarban District plus Chakaria in Cox’s Bazar District. The high rates in Fatikchari, Rangonia and Chakaria were a particular surprise as these had not been previously identified as high risk areas, although they are near to the forest fringe. Few of the affected individuals in these three areas had visited the adjacent CHT. Even though very few cases of malaria seen at CMCH lived in the CHT, this study indicated almost three times this number, over a third of the total, are likely to have become infected there. Thus the CHT are an important source of malaria both for residents and travelers, although an important proportion of malaria transmission is outside this area, as has also been described in previous studies [1]. Albeit lower transmission outside the CHT, the much larger population in this area contributes significantly to the malaria case load. In this study two thirds of cases had not visited the CHT during the time in which they became infected. Malaria control efforts to date have been particularly focused on the CHT [18] but these data suggest a broader area should be targeted.

There was a limited number of cases from Khagrachari District, the northern third of the CHT, previously found to be the area with the highest transmission in Bangladesh [9]. The small District hospital in Khagrachari town is similar to those in Bandarban and Rangamati and lacks facilities for mechanical ventilation or renal dialysis, which are often needed for patients with severe falciparum malaria. The long travel time to Chittagong might discourage families and physicians from referral to CMCH.

Another underrepresented area known to be highly malarious was Rangamati District, particularly in the east near the border with India, although this area is also an endemic zone with relatively high transmission [9]. There are a number of possible reasons for this. A previous study showed that there is a strong preference among indigenous people in this area for seeking treatment from alternative practitioners in the first instance, although this may be different for the severely ill [16]. Transport from this area to Chittagong is also difficult. Between much of this area and Chittagong city is a large man-made lake, Kaptai Lake, and this can only be crossed by a long journey by boat. Road links to this area are poor, particularly in the wet (malaria) season, and from many areas the travel is long and arduous. These difficult travel conditions might encourage people to seek treatment locally. However, patients from other remote areas with similar difficulties during the wet season did reach CMCH.

Although there are many pharmacies and health centres in the CHT [16], few of these can provide intravenous treatment and are very limited in their ability to provide more extended supportive care. There are larger and better equipped District hospitals in Khagrachari, Rangamati and Bandarban towns, but these cannot offer mechanical ventilation or renal dialysis, for which referral to a tertiary center is necessary. The dearth of referrals from the CHT to CMCH thus indicates there is likely to be a large burden of patients receiving suboptimal medical treatment in the periphery, and the perceived and real risks of long transportation times to a tertiary treatment centre are likely part of the explanation. For very remote areas in the wet season, it may be that the risk of transport is just too high and the emphasis therefore has to be on improving care locally as much as possible. One recent example has been the introduction of pre-referral treatment with rectal artesunate. This has the advantage that patients begin effective treatment earlier and could potentially ‘buy time’ to allow them to transfer more safely to a better equipped facility [19]. Expanding the availability of effective early antimalarial treatment in general, as has been occurring over the past few years [5], will also mean fewer patients progressing to severe disease.

Although overall numbers were large, this study had several limitations. All data were from a single tertiary referral hospital. Data were only collected on those patients who had a malaria test by the on-site malaria diagnostic laboratory. There are several private laboratories in Chittagong who also provide malaria tests, although they charge a fee for this service. A small proportion of patients still undergo testing by these private laboratories although the vast majority of these are retested by the hospital laboratory. The study relies on the assumption that the quality of malaria diagnosis did not change significantly from 1999–2011. This is likely to be the case, as the same highly experienced staff were employed throughout and used the same techniques. It does, however also rely on the medical staff referring the same group of patients for testing during this period but data on this were not collected. Numbers of cases from the CHT and total numbers of *P. vivax* were small. Conclusions regarding *P. vivax* epidemiology are thus limited in their scope.

Since it has been reported in the most recent government report that in 2008 3.8% of *P. falciparum* cases in Bangladesh occurred in Chittagong District [7], and we found that, in 2008, 130/165 CMCH admitted severe malaria cases were from that area, the total national number of severe malaria cases in Bangladesh was at least 130/0.038 = 3460. This figure ignores any patients with severe malaria admitted to the many other hospitals in Chittagong District, as well as those that did not reach healthcare and the actual total is thus likely to be much larger. Official data report a total number of 3591 severe cases for the whole country from mid 2008 to mid 2009, which is thus a severe underestimation [7] and there is a clear need for more accurate and complete reporting. Current systems for collating this data are incomplete and many confirmed cases are missed from the official totals [1-4]. Of particular interest would be the trends in numbers of cases, severe malaria and malaria deaths in the CHT which is essential information to assess the efficacy of malaria control measures as well as for the allocation of resources for patient care.

## Conclusions

The lack of epidemiological data on severe and fatal malaria in Bangladesh makes it difficult to plan allocation of healthcare provision to achieve maximum impact on mortality with limited resources. This study demonstrates that a very small proportion of severe malaria cases receive essential high-level supportive care. Patients from the highest transmission zone, the CHT, are particularly underrepresented. These patients could signify an important hidden group with potentially preventable mortality. Despite the recent reductions in malaria incidence and mortality, optimizing care for those developing clinical disease and especially severe malaria remains a priority. Investment in improving early treatment and simple supportive care for severe malaria at peripheral sites should be prioritized together with streamlining referral pathways to higher level care facilities.

## Competing interests

The authors declare that they have no competing interests.

## Authors’ contributions

RJM conceived of and designed the study, analysed the data and drafted the manuscript. RJM, SKP, WR, NV and RRM collected and entered the data. MUH, MAH, AAS, WR, AG, RA, RS, EBY, MGH and AMF provided clinical care for the patients. All authors read and approved the final manuscript.

## Supplementary Material

Additional file 1 **Video 1.** Map of annual number of cases of *P. falciparum* by area of residence from 2002–2011. Before 2008, data on Thana of residence were not available for those from the CHT. (Best viewed using QuickTime player (Apple Inc., CA, USA)). Click here for file
